# Advances in Therapies for Mucopolysaccharidoses

**DOI:** 10.3390/cimb48070671

**Published:** 2026-06-29

**Authors:** Joanna Szmydtka, Maja Ziemian, Rafał Banaszak, Martyna Ciesielska, Jagoda Ładosz, Urszula Maliszewska, Adrian Nowakowski, Martyna Paszek, Gabriela Suproń, Zuzanna Świętochowska, Agata Tkaczuk, Szymon Wojciechowski, Grzegorz Węgrzyn

**Affiliations:** Department of Molecular Biology, Faculty of Biology, University of Gdańsk, Wita Stwosza 59, 80-308 Gdańsk, Poland; j.szmydtka.920@studms.ug.edu.pl (J.S.); m.ziemian.400@studms.ug.edu.pl (M.Z.); r.banaszak.145@studms.ug.edu.pl (R.B.); m.ciesielska.930@studms.ug.edu.pl (M.C.); j.ladosz.582@studms.ug.edu.pl (J.Ł.); u.maliszewska.572@studms.ug.edu.pl (U.M.); a.nowakowski.744@studms.ug.edu.pl (A.N.); m.paszek.561@studms.ug.edu.pl (M.P.); g.supron.651@studms.ug.edu.pl (G.S.); z.swietochowska.937@studms.ug.edu.pl (Z.Ś.); a.tkaczuk.961@studms.ug.edu.pl (A.T.); s.wojciechowski.403@studms.ug.edu.pl (S.W.)

**Keywords:** mucopolysaccharidoses, gene therapy, enzyme replacement therapy, stem cells, glycosaminoglycans, natural compounds, substrate reduction therapy

## Abstract

Mucopolysaccharidoses (MPS) are severe, inherited metabolic diseases, classified among lysosomal storage diseases (LSDs). The presence of pathological variants of genes coding for enzymes involved in the degradation of glycosaminoglycans (GAGs) is a primary cause of each MPS type, and accumulation of these compounds is a characteristic feature of MPS. Depending on the kind of defective enzyme and the type of stored GAG(s), 12 classical types are distinguished, and a few other related diseases, whose classification is unclear. Although there is no fully effective cure for MPS, several kinds of therapeutic approaches have been proposed to treat these diseases, and some of them have been introduced into clinical practice. In this review article, we present and discuss very recent advances in developing various therapies for MPS, also indicating problems and limitations. This paper focuses on enzyme replacement therapy (ERT), cell- and gene-based therapies (including hematopoietic stem cell transplantation and gene therapy), inhibition of GAG synthesis, and some other newly developed therapeutic approaches. Perspectives on MPS therapies are also discussed.

## 1. Introduction

Mucopolysaccharidoses are a group of inherited metabolic diseases characterized by lysosomal accumulation of glycosaminoglycans (GAGs). Mutations in genes encoding enzymes responsible for GAG degradation result in impaired or completely abolished breakdown of these compounds within lysosomes. Consequently, GAGs progressively accumulate in these organelles, leading to their enlargement and dysfunction. Increasing GAG storage not only results in the accumulation of undegraded substrate but also alters the lysosomal environment, impairing the activity of other lysosomal enzymes and leading to secondary accumulation of additional cellular compounds [1]. Furthermore, increased permeability of damaged lysosomes may result in the release of lysosomal contents into the cytoplasm, activating inflammatory pathways and disrupting cellular homeostasis. As a consequence, chronic inflammation, oxidative stress, mitochondrial dysfunction, and endoplasmic reticulum stress may develop [1]. In addition, progressive GAG accumulation leads to enlargement of cells and subsequently of entire tissues and organs. These alterations contribute to the development of the characteristic multisystem manifestations of MPS, including airway narrowing, thickening, and dysfunction of cardiac valves, skeletal abnormalities, and neurological impairment [2].

According to this definition, there are 12 identified “classical” types and subtypes of MPS, and a few MPS-related diseases that can or cannot be included in the MPS group, depending on the definition and classification rules (Table 1). Nevertheless, each MPS type/subtype and each MPS-related disease is caused by the dysfunction of a separate gene. The incidence of mucopolysaccharidoses varies considerably across different countries and populations, ranging from less than 1 to as many as 16.9 cases per 100,000 live births in some regions. In addition to differences in the overall frequency of MPS, the distribution of individual disease subtypes also varies geographically. Certain MPS types may predominate in specific populations, whereas they are observed only rarely in others [3]. The phenotype in MPS depends not only on the type of mutation but also on the level of residual enzymatic activity and which enzyme is affected. Mutations leading to complete loss of enzyme activity are usually associated with a more severe disease course, earlier onset of symptoms, and faster disease progression. In contrast, mutations allowing preservation of residual enzyme activity often result in a milder phenotype and slower disease progression [4,5,6]. The stage of glycosaminoglycan degradation at which the defect occurs may also be important. Even in cases where the same substrate accumulates, differences in the affected enzyme may influence the predominance of neurological or somatic manifestations [7]. Genotype–phenotype correlations may also have clinical relevance, as they can help predict disease course and may influence the choice of therapeutic strategy.

Among MPS-related diseases, there are MPS IIIE, MPS-plus (MPSPS), and MPSPS-like syndromes. Their inclusion in or exclusion from the broad MPS family is controversial. MPS IIIE is characterized by symptoms typical of MPS occurring only in a mouse model, while expressed in humans as the Usher syndrome type IV; MPSPS is a disease caused by mutations in the *VPS33A* gene, leading to instability of the gene product and disturbances in vesicular transport, causing endosomal GAG accumulation; and MPSPS-like syndrome mimics MPSPS in symptoms but is caused by pathogenic variants of *VPS16* [12,13,22] (Table 1).

Irrespective of the MPS type, there is no fully effective cure for these diseases. Some therapies revealed partial efficacy, with efficacy significantly higher if the treatment was started at a very early stage of life (preferably before the 2nd year of life); however, diagnostic difficulties and late recognition of MPS make the effective treatment relatively unlikely under current conditions [23]. Nevertheless, some therapies are already approved and used clinically, with others being under development.

Although therapies for MPS were reviewed previously, those review articles were focused either on a specific MPS type or a specific kind of therapy. Moreover, the field of developing novel therapeutic approaches and detailed testing of already existing ones is expanding so quickly that we aimed to analyze very recent discoveries and advancements in this area. Therefore, we present a discussion on what are the newest improvements in the way to construct efficient therapies which might cure or at least significantly improve the quality of life of patients suffering from MPS.

## 2. Methods

To analyze the progress in therapies for MPS, publications from the PubMed database (https://pubmed.ncbi.nlm.nih.gov/; last accessed on 18 March 2026) from the years 2021–2026 were used. The search was conducted using the phrase “mucopolysaccharidosis and therapy”. Among the 745 publications found, 315 directly discussed therapies (others were excluded from the analysis as they simply mentioned the therapeutic possibilities or indicated that patients were treated or not with existing therapies, without a deeper analysis of the effects or mechanisms of the therapies). Following detailed analysis, 134 articles were selected for deeper analyses, and the papers excluded at the second stage were those published in a non-English language, review articles presenting mostly principles of specific therapies, papers focused on therapies for other diseases where MPS were mentioned only for comparison reasons, and essays discussing rare diseases where therapies for MPS were described only as examples. In the final list, the following articles were used for detailed analysis: 6 publications concerning ERT, 29 publications concerning HSCT, 7 publications describing gene therapies using lentiviral vectors (LVs), 29 publications concerning gene therapies related to vectors based on adeno-associated viruses (AAV), 7 publications discussing other genetic modifications, 3 publications on RNA-based therapies, 10 articles analyzing new therapeutic targets and developing treatment strategies, and 7 publications devoted to natural compounds. Additionally, 72 other publications were cited to introduce the individual chapters, as well as to prepare the introduction and discussion sections.

## 3. Enzyme Replacement Therapy

Over the years, ERT has become the mainstay of treatment for LSDs, especially for MPS. Despite its numerous advantages, such as a significant reduction in GAGs levels, a decrease in liver and spleen volume, an improvement of visceral organ function, walking ability and joint mobility, and overall enhancement of the patient’s quality of life, it has many drawbacks. ERT has a limited ability to cross the blood–brain barrier (BBB), making treatment of the central nervous system (CNS) ineffective. Currently approved intravenous ERTs for MPS include laronidase for MPS I (0.58 mg/kg once weekly), idursulfase for MPS II (0.5 mg/kg once weekly), galsulfase for MPS VI (1 mg/kg once weekly), elosulfase alfa for MPS IVA (2 mg/kg once weekly), and vestronidase alfa for MPS VII (4 mg/kg every other week) [24,25,26]. Across these disorders, long-term studies have shown sustained somatic benefit; for example, laronidase in MPS I was associated with improved mobility, joint range of motion, and reduced hepatosplenomegaly [27], whereas idursulfase in MPS II and elosulfase alfa in MPS IVA significantly improved the composite endpoint based on 6 min walk distance and forced vital capacity [26], but conventional ERT still has a limited effect on skeletal progression [28].

This therapy provides a recombinant functional enzyme to deficient cells via the mannose-6-phosphate receptor (M6PR), which targets M6P-tagged proteins to lysosomes for catabolism of accumulated substrates [29,30]. To circumvent the limitations of M6PR-mediated transport in the brain, novel fusion proteins have emerged that utilize alternative receptor pathways. Representing a significant breakthrough in this field, pabinafusp alfa is a recombinant human IDS genetically fused to the C-terminus of the heavy chain of an antibody targeting the human transferrin receptor (hTfR). In a recent work, both preclinical and clinical findings supporting the efficacy of pabinafusp alfa were summarized [31]. Preclinical studies in murine models demonstrated that intravenously administered pabinafusp alfa significantly reduces GAG levels in peripheral tissues as well as in the brain, owing to its ability to cross the BBB. Specifically, in IDS-deficient mice, pabinafusp alfa reduced HS concentrations in the brain by approximately 90% after 36 weeks of treatment, reaching levels near those of wild-type animals. In contrast, conventional ERT (idursulfase) showed no reduction in brain HS levels, resulting in a nearly 8-fold difference in substrate accumulation between the two therapies [31].

Furthermore, treatment was associated with attenuation of neuroinflammation, characterized by a decrease in GFAP-positive astrocytes and CD68-positive microglia which contributed to the prevention of progressive morphological abnormalities and neuronal loss observed in untreated animals [31]. Regarding somatic efficacy, pabinafusp alfa showed a comparable effect to conventional IDS ERT, with serum HS levels decreasing by approximately 75–80% [32].

In clinical studies, a significant reduction in GAG levels was observed in serum, urine, and cerebrospinal fluid (CSF), indicating CNS activity of the drug in patients. In a Phase II/III trial, patients treated with pabinafusp alfa achieved a mean reduction of 71.3% in CSF HS concentrations after 52 weeks, whereas conventional ERT typically has no measurable impact on CSF biomarkers [31]. These biochemical effects were accompanied by signals of improvement or stabilization in neurocognitive function [33]. This therapy is available mainly in Japan, but in March 2026, the FDA approved an ERT, tividenofusp alfa-eknm (Avlayah), for the neurological manifestations of MPS II. This therapy involves intravenous administration of IDS fused to a TransportVehicle platform. The Fc domain of the fusion protein binds to the TfR, enabling receptor-mediated transcytosis across the BBB and delivery of the enzyme to the CNS [10]. Clinical trials demonstrated that at 24 weeks, tividenofusp alfa achieved a mean reduction of 91% in CSF of HS, with 93% of patients reaching levels within the normal range of unaffected children [34]. Furthermore, long-term data revealed a 70,5% reduction in serum neurofilament light (NfL) levels by week 104, suggesting a significant attenuation of neuroaxonal injury. These biochemical improvements were correlated with clinical benefits, including stabilization or gains in adaptive behavior and a mean improvement of +5.9 points on the Bayley Scales of Infant and Toddler Development (BSID-III) [34] (https://www.neurologylive.com/view/fda-grants-accelerated-approval-to-tividenofusp-alfa-for-neurologic-hunter-syndrome; accessed on 2 May 2026).

Overall, every MPS needs an individual approach to meet the patient’s requirements. Many therapies still need to be worked on to optimize their potential. There are many ERT approaches being conducted, but it is important to consider that combined therapy may be even more beneficial and may resolve many complications connected with ERT limitations.

## 4. Cell- and Gene-Based Therapies

### 4.1. Introduction to Cell- and Gene-Based Therapies for MPS

**Figure 1:**
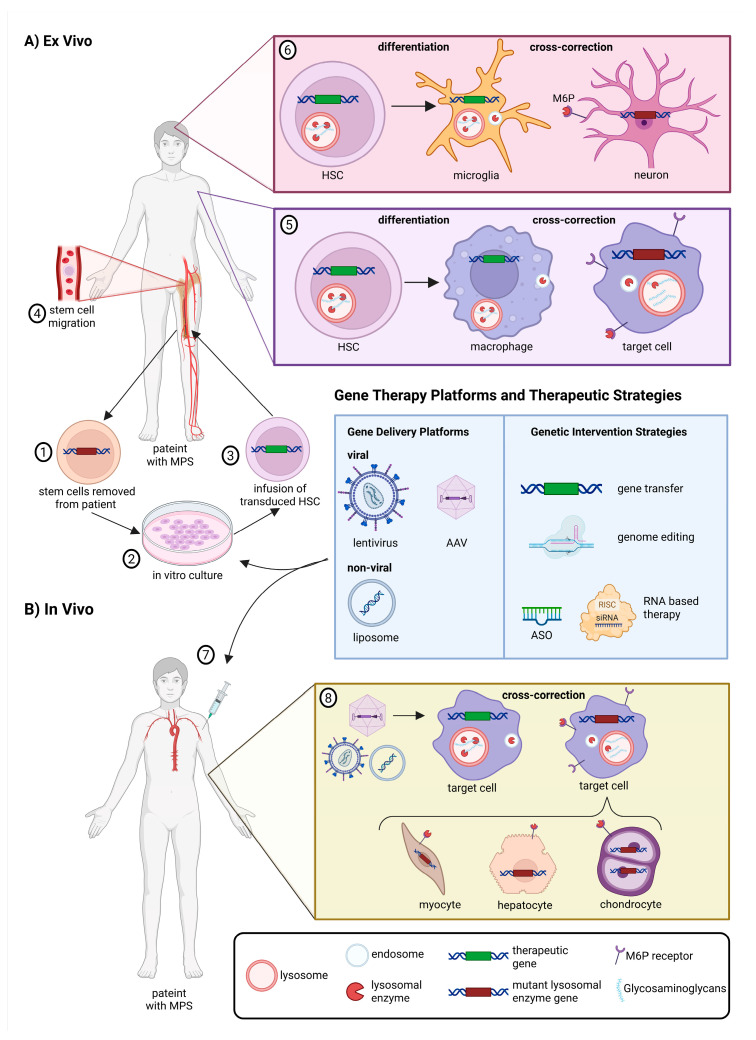
Strategies of ex vivo and in vivo gene therapies in MPS. (**A**) Ex vivo Gene Therapy: (1) HSPCs are collected from the patient via bone marrow aspiration or peripheral blood mobilization. (2) Isolated cells are cultured in vitro and genetically modified mainly using LV for stable gene integration or precision genome editing (e.g., CRISPR-Cas9/AAV-mediated HDR). (3) The transduced HSPCs are re-infused into the patient following a conditioning regimen. (4) These cells migrate via the bloodstream and (5) differentiate into tissue-resident macrophages in peripheral organs or (6) cross the BBB to differentiate into microglia-like cells. Through cross-correction, the corrected cells secrete functional lysosomal enzymes, which are internalized by enzyme-deficient target cells via M6PR, effectively reducing GAG accumulation. (**B**) In vivo Gene Therapy: (7) Therapeutic payloads, including AAV vectors carrying the transgene or genome-editing machinery (CRISPR-Cas, ZFNs), are administered systemically or locally. (8) Transduced cells in “depot organs” such as the liver, or local tissues (myocytes, chondrocytes, hepatocytes), continuously produce and secrete the functional enzyme into the circulation. Additionally, non-viral platforms (e.g., liposomes) can be utilized for siRNA-based substrate reduction therapies or antisense oligonucleotides (ASO) to silence the RNA. Created in BioRender. Ziemian, M. (2026) https://BioRender.com/xan1jz3, accessed on 2 May 2026.

There are therapeutic approaches (both approved and those remaining in clinical and preclinical phases) based on delivering a correct copy of the gene to the body, editing the gene, as well as administering cells expressing a functional gene. Therapies involving the transplantation of hematopoietic cells producing the correct enzyme were among the first strategies to attract significant clinical interest and, to this day, remain one of the main therapeutic options in the treatment of MPS I and MPS II. However, due to the dynamic development of alternative treatment methods, this approach is increasingly considered as a component of combination therapy. It may contribute to prolonged survival and slowing of symptom progression, but is less frequently regarded as a standalone, definitive therapeutic solution [11]. Currently, a more dynamic development of gene therapies than HSCT itself is being observed. Gene therapies can be divided into ex vivo and in vivo approaches (Figure 1) [35]. The ex vivo strategy involves modifying the patient’s HSC outside the body and subsequently re-administering them after appropriate genetic processing. Initially, gamma-retroviral vectors were used in this approach; however, LVs are now predominantly used due to their higher efficiency and safety profile. The use of the patient’s own genetically modified cells eliminates the risk of immunological graft rejection, which was a significant issue in classical HSCT [35]. Ex vivo strategies also include various forms of genome modification using tools such as transcription activator-like effector nucleases (TALENs), zinc finger nucleases (ZFNs), or the Clustered Regularly Interspaced Short Palindromic Repeats (CRISPR) with CRISPR-associated proteins (Cas), in which AAV vectors are used as carriers of the editing system components or DNA repair template. However, AAV vectors are primarily used in in vivo approaches, which involve the direct delivery of a transgene into the body, where it most often persists in the form of episomal DNA [36]. Therapeutic strategies based on oligonucleotides are also being developed to modify gene expression (e.g., to reduce GAGs accumulation or to prevent premature stop codons) [37,38].

Despite these major advances, gene therapy for MPS still faces several important challenges. Vector immunogenicity remains a major concern, particularly for AAV-based in vivo approaches, because pre-existing neutralizing antibodies and adaptive immune responses may reduce transduction efficiency or limit repeat dosing [35,39]. In addition, the durability of therapeutic expression is not always sufficient, especially in dividing tissues, where episomal AAV genomes may be progressively diluted. Integrating vectors and genome-editing platforms may provide longer-lasting correction, but they also raise concerns regarding insertional mutagenesis, off-target effects, and long-term genomic safety. Another major limitation is manufacturing complexity, as the production of clinical-grade viral vectors requires highly controlled GMP conditions, extensive quality testing, and remains costly and time-consuming [40]. Finally, regulatory approval is further complicated by the need for prolonged safety monitoring, careful assessment of genotoxicity, and standardized evaluation of potency, biodistribution, and immune responses. The detailed development of individual therapeutic strategies is presented in the subsections below.

### 4.2. Haematopoietic Stem Cell Transplantation

#### Introduction to HSCT in MPS

The rationale for HSCT is based on the mechanism of cross-correction. Donor-derived cells differentiate into cells of the mononuclear—macrophage system and produce the missing enzyme, which can subsequently be taken up by recipient cells. This mechanism enables systemic enzyme delivery and partially affects the CNS. Donor-derived cells can migrate to the brain and differentiate into microglia (Figure 1). Although neurological function is not completely restored, this process provides a biological basis for the use of HSCT in neuronopathic forms of MPS, as demonstrated in studies on MPS I and MPS II [41,42].

The greatest clinical experience with HSCT concerns the severe form of MPS I. Long-term observations spanning several decades confirm that HSCT is an established treatment for this condition [41]. Advances in transplantation include improved conditioning regimens with pharmacokinetically controlled busulfan dosing, the use of different stem cell sources such as bone marrow and umbilical cord blood, and studies evaluating factors affecting engraftment and transplant failure [43,44,45]. Long-term follow-up studies have also assessed treatment outcomes and late complications [41,46]. Due to this extensive experience, MPS I has become a reference model for evaluating HSCT in other metabolic disorders. Both single-center and multicenter studies demonstrate improvements in transplantation safety and outcomes over time [47,48]. In contrast, HSCT in MPS II, MPS IVA, MPS VI, and MPS VII remains more selective and is mainly considered in severe phenotypes or cases with significant neurological involvement (Table 2) [49,50,51,52].

Gene therapy using autologous CD34+ cells represents an evolution of traditional HSCT. A translational study in MPS II demonstrated long-term enzyme expression and multisystem improvement using genetically modified autologous cells [61]. This strategy avoids GVHD and eliminates the need for HLA matching while preserving the ability of myeloid cells to migrate to the CNS. Allogeneic HSCT depends on donor availability; HLA-identical sibling donors remain the preferred option, yet they are available for only about 25% of patients [62]. Gene therapy has emerged as a promising alternative to conventional allogeneic HSCT for MPS. This strategy is based on transplantation of autologous CD34+ hematopoietic stem and progenitor cells (HSPCs), thereby eliminating the need for donor identification and HLA matching. Over recent years, numerous preclinical studies have explored gene therapy in HSCT approaches using CRISPR-based genome editing, integrase-deficient LV, TALEN-mediated gene correction, nanoparticle delivery systems, and promoterless gene-targeting strategies. Nevertheless, ex vivo modification of CD34+ cells using LVs has become the dominant platform because of the stable integration of the therapeutic transgene into the host genome, enabling long-term hematopoietic reconstitution. Currently, several clinical studies for MPS are underway, all employing lentiviral-based gene transfer approaches. In MPS IH, clinical studies using autologous CD34+ cells transduced with an LV encoding the *IDUA* gene (https://clinicaltrials.gov/studyNCT06149403, accessed on 2 May 2026) demonstrated sustained supraphysiological IDUA activity in peripheral blood, reaching 0.7–18-fold above the mean activity observed in healthy individuals, accompanied by a marked reduction in urinary GAG excretion by approximately 89–90% within 12 months [63]. Furthermore, corneal clouding completely resolved in 37.5% of treated patients, whereas none of the patients treated with conventional allogeneic HSCT achieved complete resolution, with at least moderate corneal opacity persisting in all cases [64]. In neuronopathic MPS II, an ongoing study (https://clinicaltrials.gov/studyNCT05665166, accessed on 2 May 2026) employs autologous CD34+ HSPCs transduced ex vivo with a CD11b-directed lentiviral vector encoding human IDS fused to an ApoEII peptide designed to enhance CNS delivery. Early clinical results demonstrated supraphysiological IDS activity in plasma and leukocytes persisting for at least three months after treatment, together with progressive increases in IDS activity in CSF and bone marrow. In parallel, urinary GAG concentrations continued to decline toward the normal reference range [65]. In MPS IIIA (Sanfilippo syndrome type A), treatment with autologous CD34+ cells transduced with a lentiviral vector expressing SGSH under the control of the CD11b promoter (https://clinicaltrials.gov/studyNCT04201405, accessed on 2 May 2026) resulted in supranormal SGSH activity in leukocytes one month after infusion, reaching levels 38–91-fold higher than the median normal value. SGSH activity in CSF normalized or exceeded normal values up to month 6, while HS concentrations decreased by more than 90% in urine and more than 82% in plasma [66].

In addition to ongoing clinical studies, numerous preclinical investigations continue to evaluate LV-HSCT approaches for other MPS subtypes, including MPS IVA. To address the persistent challenge of skeletal dysplasia in MPS IVA, transplantation of autologous HSCs modified with lentiviral vectors expressing GALNS under either the CBh or COL2A1 promoter resulted in partial correction of skeletal abnormalities in MPS IVA murine models, although enzyme activity remained below physiological levels. Notably, complete correction of cardiac pathology was achieved, suggesting that further optimization of vector design and conditioning regimens may improve overall therapeutic efficacy [67]. Beyond lentiviral GT-HSCT, the preclinical MPS field has also explored several alternative hematopoietic stem cell engineering platforms, including IDLV-assisted CRISPR/Cas9 knock-in approaches for MPS IVA, and non-viral magnetite-based nanoparticle delivery of CRISPR/nCas9 in MPS IVA fibroblast models [68,69]. In parallel, TALEN-based intron editing has been developed as an HSPC engineering strategy for myeloid-restricted transgene expression in MPS I [70]. Promoterless CRISPR/Cas9 gene targeting has also shown proof-of-concept efficacy in an MPS VII mouse model using a high-capacity adenoviral platform [71].

Currently, allogeneic hematopoietic stem cell transplantation (allo-HSCT) remains the standard therapeutic approach in MPS. In economic terms, allo-HSCT is generally less expensive than autologous HSCT-based gene therapy, largely because it does not involve costly ex vivo gene-modification procedures. Although the overall cost of allo-HSCT may exceed that of enzyme replacement therapy (ERT) in the United States, it may still represent a more advantageous strategy due to its one-time nature and the potential for durable endogenous enzyme production, eliminating the need for repeated weekly infusions. Moreover, HSCT appears to provide better correction of certain manifestations, especially those affecting the central nervous system [72]. However, both therapeutic modalities entail significant clinical risks. Moreover, HSCT is primarily associated with graft-versus-host disease (GvHD), graft rejection, and toxicities linked to intensive myeloablative conditioning. Conversely, autologous HSCT-based gene therapy, while circumventing GvHD, poses risks related to the efficiency of ex vivo transduction, durability of transgene expression, and the potential for insertional mutagenesis leading to oncogenesis [73,74].

Overall, HSCT shows its most established benefit in severe MPS I, is used more selectively in MPS II, and remains less certain in MPS IVA and VI, where skeletal and corneal manifestations are often only partially corrected. It is also worth noting that timing is critical, as HSCT is most effective when performed early, ideally before 24–30 months of age and before major neurocognitive impairment develops, especially in severe MPS I [75].

### 4.3. Lentivirus-Based Therapies

Retroviruses belong to the family of Retroviridae, including the genus Lentivirus (International Committee on Taxonomy of Viruses (ICTV), https://ictv.global/taxonomy; accessed on 2 May 2026). They are RNA viruses that encode reverse transcriptase, which allows viral RNA to be transcribed into double-stranded DNA and integrated into the host genome. This latter property can be an advantage over other viral vectors such as AAV, because integration enables long-term transgene expression without the “vector dilution effect” that can occur with other vectors during cell proliferation [76]. Within Retroviridae, LVs are used far more often than gamma-retroviral vectors: lentiviruses can transduce non-dividing cells, whereas integrating retroviral vectors typically require mitosis because they cannot efficiently traverse the nuclear envelope to integrate into the host genome [76,77].

Lentiviral vectors are predominantly employed in ex vivo gene therapies. This approach allows for selective, long-term, and controlled cellular modification, which minimizes the risks of off-target effects, oncogenesis, and immune activation triggered by the viral capsid [77,78]. However, ex vivo therapy faces significant hurdles: the patient must undergo a rigorous conditioning regimen (chemotherapy) before transplantation, leading to numerous adverse effects. Furthermore, the process is logistically complex, requiring specialized laboratory infrastructure [79]. For MPS IVA, HSCT remains an investigational option rather than a recommended standard of care, as current evidence is limited and does not support routine use. Although HSCT can increase GALNS production and thus enable some cross-correction, it has not produced improvements in growth or skeletal dysplasia; late timing of the intervention may also have contributed to the lack of skeletal benefit [80].

Puentes-Tellez et al. investigated an in vitro approach using a lentiviral vector carrying *GALNS* and *SUMF1* cDNAs delivered to fibroblasts from MPS IVA patients. *SUMF1* was included because elevated levels of formylglycine-generating enzyme (FGE) improve the conversion of cysteine to formylglycine, increasing the fraction of properly activated GALNS molecules and thereby raising enzymatic activity for a given amount of GALNS protein. This therapy restored GALNS activity, reduced lysosomal mass, and affected the autophagic pathway in patient cells [81].

Celik et al. explored in vivo delivery of a lentiviral vector encoding GALNS to MPS IVA mice using either the ubiquitous Cbh promoter or the cartilage-specific COL2A1 promoter; this followed an ex vivo approach that did not produce the expected results [67]. Intravenous administration increased GALNS levels and reduced GAG accumulation. The study also demonstrated that in vivo lentiviral therapy can ameliorate bone pathology, but efficacy depended on early intervention [57]. To address the difficulty of achieving therapeutic GALNS levels in bone, capsid modifications have been investigated—such as incorporating the WYRGL peptide into the capsid ligand, which enhanced the vector’s affinity for collagen within the cartilage of MPS IVA mice [82]. Although in vivo lentiviral administration is not yet the clinical standard, it remains a promising alternative for targeting specific tissues in metabolic disorders [83].

### 4.4. Adeno-Associated Virus-Based Therapies

AAV vectors are becoming an increasingly popular therapeutic strategy due to several key advantages. These include the persistence of the transgene mainly in an episomal form, resulting in a very rare integration into the host genome (thus avoiding a risk for inactivation of an off-target gene), and the lack of replicative capacity of the recombinant vector in the body [77]. Additionally, AAV is characterized by a wide range of serotypes, enabling precise targeting of therapy to particularly affected tissues, as well as the possibility of further modifications of the capsid and genetic construct, allowing for even greater specificity and efficacy of action [36,77]. Currently, there are no approved gene therapies based on AAV vectors for any type of MPS. However, in recent years, several clinical trials have been published that have provided important information regarding the efficacy and safety of this therapeutic strategy.

In a phase 1/2 clinical trial concerning MPS VI, a clear dose-dependent effect was demonstrated. Only the highest administered dose enabled maintenance of stable enzyme and GAG levels in the body during long-term follow-up. Moreover, no significant decline in transgene expression over time was observed in young patients, suggesting that the risk of episomal DNA dilution in the liver may be lower than originally assumed [84]. In turn, from the phase 2/3 clinical trial for MPS IIIA, it can be concluded that, in addition to the type of vector and promoter, the choice of the appropriate delivery technique is also crucial. In that study, following brain injection, local enzyme overproduction was observed, which led to disturbances in tissue homeostasis at the injection site, including depletion of physiologically occurring HS. Although in the analyzed case the changes were not permanent, the procedure was performed in a very young patient, so the regression of changes may have been related to greater neural tissue plasticity at the specific stage of life. The impact of similar overexpression of the recombinant gene on more mature tissue remains unknown [85]. Researchers conducting a phase 1/2 clinical trial for MPS IIIB demonstrated that the body cannot develop immune tolerance to the administered enzyme if it was not previously produced endogenously. Throughout the entire study period, waves of NAGLU-specific T lymphocytes were observed. At the same time, stable enzyme production suggested that appropriately implemented immunosuppression may be key to maintaining the therapeutic effect. Moreover, those results highlighted how significant early therapeutic intervention is, as the clinical effect of the therapy was maintained throughout the study duration only in the youngest patient [86]. Similar conclusions arose from an uncontrolled phase 1/2 trial, also for MPS IIIB, in which only the youngest patient showed no progression of brain atrophy and had the mildest immune response to the therapy [87].

Despite the limited number of clinical trials, between 2021 and 2026, the AAV strategy was intensively developed in numerous preclinical studies. One of the key aspects that received attention is the selection of an appropriate vector in terms of tropism. Individual types of MPS differ in their organ involvement profile, as in some cases the disease is multisystemic, whereas in others, changes predominantly affect specific tissues. Preclinical studies have shown that AAV9 is characterized by broad tissue tropism, including bones and the CNS [88,89]. In contrast, AAV8, due to its strong liver tropism, is often used to achieve significant circulating enzyme concentrations so that the therapy can act systemically [90,91,92].

An interesting relationship between sex and tropism was also observed, as in one study, AAV9 showed more efficient cell transduction in male organisms than in females. It was suggested that testosterone may be responsible for the observed differences [93]. To optimize the therapy, researchers also focused on the impact of dose on the final outcome. In most analyses, there were the highest administered doses that led to a clear and sustained therapeutic effect in various types of MPS [93,94,95,96]. At the same time, dose remains an element requiring particular caution. It was demonstrated that females are characterized by a more reactive immune response to therapies and have more enzyme-neutralizing antibodies [97]. Consequently, increasing the dose may paradoxically weaken the therapeutic effect [97]. In another report, attention was drawn to the possibility of reducing the vector dose through combination therapy. Instead of increasing the amount of administered AAV to obtain a stronger therapeutic effect, it was proposed to combine gene therapy with additional strategies targeting secondary disease mechanisms. Researchers applied AAV therapy in combination with the small molecule CLR01, which reduced amyloid deposition and alleviated secondary pathologies [98].

Combination therapy was also applied in MPS IVA, where, in addition to AAV8 therapy, a bone growth-stimulating peptide was used. However, this effect did not lead to full correction of deformities (curvatures elongated along with the bone). Thus, it can be concluded that the effectiveness of therapy is strongly dependent on the timing of its initiation, as late intervention may not reverse existing deformities and, in the worst case, may modify their further progression. Another equally important element in therapy design that has been observed is the selection of an appropriate promoter. It was demonstrated that the use of the CAG promoter led to a better therapeutic effect in MPS IVA than a fourfold higher dose of AAV with the TBG promoter [99]. In other preclinical studies, attempts were also made to modify the transgene product itself in order to increase its tissue efficacy [100]. In some strategies, AAV8 was used due to high enzyme production level in the liver, thereby alleviating somatic changes and, through additional enzyme modifications such as the addition of a targeting peptide, increasing its ability to reach less accessible sites such as the brain [92,101].

In the case of certain types of MPS, attempts were made to develop more complex therapeutic strategies. An example is MPS IIIC, in which the enzyme is transmembrane and does not undergo cross-correction, meaning that it cannot be effectively taken up by neighboring cells. In the study by Bobo et al., the transgene construct was modified by adding a zip code (extracellular vesicles packaging signal), enabling the packaging of the resulting mRNA into vesicles and its transport to neighboring cells [102]. Other authors also optimized the delivery technique, aiming for more uniform vector distribution within the brain to avoid expression exclusively at the injection site [103]. They used the CED technique and specialized catheters, minimizing fluid reflux [103].

Several studies also focused on optimizing routes of vector administration, as it was observed that the choice may significantly influence the distribution of expression across different tissues. In an MPS IIIA model, it was demonstrated that administration into the CSF ensured high expression in the brain and in the central part of the retina, but was less effective in the peripheral retina. In contrast, intravenous administration led to weaker brain transduction but enabled more uniform expression throughout the retina [104]. This means that the choice of administration route should consider not only maximizing the effect on one organ, but also potential consequences for other structures. Belu et al. observed that application into the brain ventricles led to the most extensive expression throughout the brain, and administration into the CSF resulted in expression mainly in the posterior part of the brain, whereas the intranasal route yielded the most limited distribution. Importantly, despite differences in enzyme levels and sites of recombinant gene expression, all three routes of administration prevented deterioration of cognitive function, suggesting that even partial biochemical correction may have significant functional relevance [105]. However, the effect within the same area depends on the type of MPS, as a similar percentage of enzyme activity might not be sufficient in every type of the disease.

Preclinical studies also focused on the modification of the AAV capsid, as attention was drawn to the fact that some amino acids present in capsid proteins may undergo phosphorylation, which promotes vector degradation and limits transduction efficiency [106]. Another important aspect discussed in the literature is the difference between research models. In the study by Chen et al., it was shown that a therapy effective in a mouse model did not produce comparable effects in macaques, highlighting the limitations of individual experimental models [95].

Taken together, these observations show that the design of AAV-based gene therapy is a multifaceted process requiring simultaneous consideration of serotype selection, promoter choice, dose, route of administration, transgene modification, and capsid properties. As therapeutic effects may vary depending on the vector used, the table below compares the AAV vectors used in MPS therapies (Table 3).

### 4.5. Gene Editing-Based Therapies

Another possibility of potential therapy that could result in phenotype correction in monogenic diseases, such as various types of MPS, is gene editing. This method uses mechanisms able to perform a double-strand break (DSB) or single-strand break (SSB), resulting in integration of a correct gene (donor gene). The most popular approach used for gene editing is CRISPR/Cas. The mechanism by which editing is performed consists of three steps. sgRNA guides the Cas9 enzyme to complementary DNA, where the enzyme can perform DSB, three base pairs upstream of the potospacer adjacent motif (PAM), creating blunt ends, and allowing for gene insertion or DNA repair [116]. This approach is widely used in cooperation with viral or nonviral vectors as a potential therapy for various types of MPS. This allows for packing the CRISPR-Cas9 cassette along with a donor gene or in two separate vectors due to the limited size of packaging [58,69,71,117,118]. It was examined whether this potential therapy could recover the correct phenotype in mouse models of MPS I [118], MPS IV [58], and MPS VII [71]. Studies with an MPS I model demonstrated an increase in IDUA activity and reduction in GAG storage in visceral tissues; however, without effect in brain tissue after intravascular injection of the therapeutic constructs to mice [119]. Hence, a new method of nasal delivery or CRISPR-Cas9, along with *IDUA* cDNA coupled in liposomes, was proposed, which was more successful in brain tissue correction. These results showed a great potential due to the efficiency of increasing the enzyme level. However, it appears that to fully normalize the GAG level, further studies must be conducted [118].

In another approach, the use of CRISPR-nCas9 and donor GALNS gene was examined, with both elements coupled with iron oxide nanoparticles (IONPs) for nonviral delivery [58]. In that study, nCas9 was used, which is a modified enzyme that has a nicking function resulting in the appearance of a single-strand break. This approach allows for higher specificity, but requires two sgRNAs to cause DSB [120]. This resulted in an increase in enzyme levels and partial correction of the phenotype in the mouse model. It was also suggested to combine donor enzyme with CRISPR-Cas9 machinery in one high-capacity adenovirus (HCAd) vector, resulting in GUSB enzyme activity in vivo and reduction in lysosomal storage even in brain tissue, proving that HCAd/Cas9-donor may reduce MPS VII phenotype [71]. Additionally, CRISPR-Cas9 can be used to perform gene correction by inducing non-homologous end joining (NHEJ) or homologous directed recombination (HDR). This method is somewhat troublesome due to the requirement of a homologous sequence for HDR to occur, thus resulting in a high level of NHEJ repair, which is not desirable in gene correction. However, a system with Nme2Cas9 packed into one AAV vector along with sgRNA and HDR template was tested, which resulted in high efficiency of gene editing in the MPS I mouse model [121]. Even though quiescent cells lean towards the NHEJ repair mechanism, resulting in low levels of HDR, in MPS I, clinically significant effects are observed even at low levels of WT protein production, thus showcasing this approach as promising for further research. The use of CRISPR-Cas9 in combination with different methods gave promising results, showing significant enzyme activity in MPS diseases, as well as reducing the phenotype. However, certain issues, such as efficient delivery through BBB, equal distribution throughout the body, immune response, or potential off-target effects, must be further studied to establish a potential therapy with the CRISPR/Cas9 system. Moreover, gene editing may be performed with machinery other than CRISPR/Cas9, such as ZFN. This approach was already clinically tested with two different groups consisting of MPS I and MPS II patients [122].

Both groups were treated with three AAV vectors packed with ZFN and donor gene, targeted for liver expression and secretion of WT protein into the bloodstream. Clinical studies indicated the safety of this therapy, as all patients did not experience severe treatment-related adverse events, with acute reactions being mostly mild and only at the highest doses of AAV. Unfortunately, genome editing was not efficient, leading to no long-term sustained enzyme expression, thus not providing therapeutic effects [122].

Recent genome-editing advances have shifted beyond nuclease-induced DSBs toward base editing and prime editing, which enable more precise correction of pathogenic variants and reduce dependence on HDR-based repair. In MPS-IH, prenatal adenine base editing has already shown proof-of-concept efficacy in a mouse model, correcting the disease-causing Idua W392X mutation in multiple organs and improving metabolic, musculoskeletal, and cardiac phenotypes [123]. Prime editing is also emerging as a versatile “search-and-replace” strategy that can introduce small substitutions, insertions, and deletions without requiring DSB, making it an attractive future option for selected monogenic MPS variants [124].

Those studies provided valuable data and knowledge that indicated further directions of research, bringing us closer to finding potential therapeutics. MPS is a monogenic disease, which makes gene editing a suitable option for potential therapy, but more studies are needed to evaluate the safety and efficiency of these methods.

### 4.6. RNA-Based Therapies

Another interesting approach for potential therapy in MPS is directly influencing mRNA, allowing for modifications of gene expression without interference with a patient’s genome. Employing siRNA or ASO, it is possible to reduce the expression level of certain genes by degradation of complementary mRNA sequences [125,126]. It was proposed to use a potentially novel approach for treatment of MPS I, based on in vitro cells. MPS I caused by nonsense mutations could be reversed by skipping the premature termination codon (PTC), allowing for full-length protein synthesis. Thus, siRNA specific for eRF3 (coding for a translation termination protein) was tested, as well as its potential in PTC readthrough in MPS I [126]. After sole treatment with siRNA, PTC readthrough was not spectacularly effective; however, in combination with aminoglycosides, higher levels of proteins were observed in the MPS I cell line [38]. Importantly, this approach increased α-L-iduronidase activity to over 3% of normal levels, a level associated with attenuated MPS I phenotypes, which was promising for further research. Nevertheless, the therapeutic use of aminoglycosides remains challenging due to their potential toxicity [38]. Another study focused on RNA silencing properties of ASO; however, in different matters. Instead of PTC readthrough, it was proposed to silence the *XYLT1* gene, as it encodes an enzyme involved in the first step of HS biosynthesis [37]. The experiments were performed with MPS IIIC fibroblasts, showing successful silencing of *XYLT1* and reducing the HS level in cell lines. This siRNA treatment needs to be further studied in in vivo disease models to fully understand its potential therapeutic value. However, it could potentially act not only for MPS III, but also for other types of MPS due to a reduction in HS biosynthesis. This approach may be particularly promising due to the translational potential of ASOs, which offer more efficient delivery to the central nervous system compared to siRNA-based therapies that often require additional carriers. Furthermore, ASOs provide controlled and reversible gene silencing rather than permanent gene inactivation, which may be advantageous given the adverse effects reported following complete suppression of XYLT1 expression [37].

A novel approach, called LEAPER 2.0, was developed, facilitating endogenous ADAR proteins to cause RNA editing by replacing adenosine with inosine [127]. This method was tested in vitro as well as in vivo in previous studies [128], proving that it efficiently decreased levels of GAGs as well as restored catalytic properties of IDUA in the MPS I mouse model. The efficiency of the phenotype correction was then tested in non-human primates and humanized mice. Again, high levels of editing were demonstrated, proving the high therapeutic value of this mechanism [127].

In summary, RNA-based therapeutics allow for various strategies in potential phenotype recovery, as could be seen in recent studies. Some methods have already been tested clinically in different diseases, which could speed up the process of accepting such therapy. Furthermore, these techniques have a wide range of effects, which could be useful to treat patients with different types of MPS. However, certain RNA-based therapies require constant dosage instead of a single one, as is the case in gene editing.

A comparative synthesis of the gene therapy approaches discussed above, highlighting their major advantages and disadvantages, is provided in Table 4.

## 5. Natural Compounds as Modulators of GAG Biosynthesis

### 5.1. Introduction to Natural Compounds for MPS Treatment

The limitations of standard MPS therapies underscore the need for alternative strategies capable of modulating disease pathology independently of lysosomal enzyme delivery while offering improved tissue accessibility. One alternative strategy is substrate reduction therapy (SRT), which targets the inhibition of GAG biosynthesis. SRT operates on the principle that reducing the rate of substrate synthesis can restore balance between GAG production and degradation, even when lysosomal enzyme activity is severely compromised [129].

This approach aims to decrease the production of GAGs, which, once synthesized in excessive amounts, accumulate within lysosomes and disrupt cellular metabolism. At the molecular level, GAG biosynthesis is initiated by glycosyltransferases that sequentially add monosaccharide units to a core protein, forming proteoglycans; targeting the regulatory pathways upstream of these enzymes therefore represents a rational therapeutic strategy [129]. By lowering the overall substrate burden, SRT facilitates more efficient degradation of GAGs by the residual lysosomal enzymatic activity due to the reduced quantity of stored material [130,131]. This therapeutic concept has stimulated interest in low-molecular-weight compounds that are capable of penetrating biological barriers like the BBB while modulating intracellular signaling pathways involved in GAG synthesis without directly disrupting other metabolic processes [132,133,134].

### 5.2. Genistein

One of the most extensively studied compounds in this context is genistein, an isoflavone predominantly found in the seeds of leguminous plants and classified as a phytoestrogen. Genistein has been investigated as a bioactive molecule with therapeutic potential in LSDs characterized by excessive GAG accumulation [135].

Genistein decreases GAG synthesis by acting as a tyrosine kinase inhibitor. It exerts its effect through modulation of the epidermal growth factor receptor (EGFR) signaling pathway, where it inhibits EGFR autophosphorylation [136]. Mechanistically, EGFR activation normally triggers a phosphorylation cascade involving the Ras/MAPK and PI3K/Akt pathways, which ultimately upregulates the expression of genes encoding enzymes essential for GAG chain elongation, including xylosyltransferases and chondroitin sulfate synthases [129,137]. This inhibition leads to downregulation of genes encoding GAG synthetases, thereby reducing substrate production. Moreover, genistein influences lysosomal biogenesis by promoting this process through modulation of transcription factor EB (TFEB), a master regulator of lysosomal function [138,139]. It also proved to induce autophagic pathways [140,141].

The ability of genistein to attenuate GAG accumulation has been demonstrated in cultured fibroblasts derived from patients with MPS I, II, IIIA, IIIB, and VII, where a reduction in urinary GAG levels has been observed following genistein [142,143,144,145]. The preclinical evidence supporting the substrate-reducing properties led to the proposal of the evaluation of this method in clinical studies [146].

Studies with an animal model of MPS II further supported its therapeutic relevance [147]. In a murine model of MPS IIIB, orally administered genistein led to reduced GAG levels, accompanied by decreased neuroinflammation, as well as correction of behavior [148]. Early clinical experiments suggested some benefits resulting from the use of genistein in MPS III patients [149]. An open-label pilot study in MPS III patients demonstrated that high-dose genistein aglycone (150 mg/kg/day) was well tolerated and associated with stabilization of cognitive decline in a subset of patients over a 12-month period [150]. That observation was corroborated in studies with a cohort of patients with MPS II, where genistein was reported to exert beneficial effects on connective tissue and joint function, leading to improvements in joint mobility and elasticity [151].

However, the overall clinical utility of genistein remains limited by the variability and ambiguity of the reported outcomes, which currently preclude definitive conclusions regarding its clinical applicability or incorporation into standard therapeutic regimens. While genistein consistently reduces GAG levels in fibroblasts, the most commonly used cellular model in MPS research, it also decreases GAG accumulation in hepatocytes, albeit to a lesser extent than in fibroblasts. In contrast, in chondrocytes, genistein has been reported to increase GAG levels, indicating cell type-dependent variability in [152].

Another major limitation concerns its efficacy in addressing neurological manifestations of MPS. In double-blind, randomized, placebo-controlled clinical trials involving patients with MPS III, genistein administration led to a reduction in GAG levels but did not result in measurable improvement in neurological outcomes [139]. A discrepancy between the high efficacy of genistein in cellular and animal models of MPS and a failure to demonstrate clinical improvement in clinical trials with MPS III patients remains to be elucidated. One might propose hypotheses that suggest a low number of patients involved in clinical trials and their high variability in age and the stage of the disease as the major causes of the failures in demonstrating clinical efficacy. Another possibility is that genistein might be effective when administered at the presymptomatic stage of the disease or at early symptomatic stages, but not when various disorders of different tissues and organs are advanced.

Recently, another activity of genistein—inhibition of proteasomal degradation—was tested in combination with ambroxol (causing a decrease in efficiency of protein degradation by ERAD) in a cellular model of MPSPS [153]. This approach was found effective in reducing GAG levels and normalizing some other biochemical parameters, including the abundance of the mutant VPS33A protein, which is otherwise severely decreased in this disease.

### 5.3. Other Natural Autophagy Modulators

Resveratrol is a widely documented polyphenol in the context of MPS research. In cellular models of Sanfilippo subtypes, this compound has been shown to promote the breakdown of stored GAGs. This effect is achieved through the activation of autophagy, specifically via an mTOR-independent pathway. Resveratrol activates SIRT1, a NAD+-dependent deacetylase, which in turn deacetylates and activates downstream targets, including PGC-1α and FOXO transcription factors, promoting autophagosome formation and lysosomal clearance [154,155]. In vivo experiments using an MPS IIIB mouse model revealed that long-term supplementation returned urinary GAG levels to normal [140]. Recent studies further demonstrate that resveratrol induces activation of TFEB and FOXO3, contributing to enhanced autophagic flux and exerting indirect anti-inflammatory effects associated with modulation of inflammatory signaling pathways. These effects are related to reduced neurodegeneration and improved behavioral outcomes in MPS IIIB mouse models [154]. Despite promising preclinical results, resveratrol faces significant translational challenges related to poor bioavailability and rapid metabolism, which have limited its progression to clinical trials in MPS [156].

Trehalose is a natural disaccharide recognized for its neuroprotective and cytoprotective properties. A noticeable decline in GAG accumulation within the brain and spleen was observed following a six-month period of oral trehalose supplementation in the MPS II model. The compound also mitigated cellular irregularities. In addition, animals treated with trehalose showed marked improvements in both mental performance and motor skills [153].

Research on the MPS IIIC mouse model indicates that glucosamine can function as a pharmacological chaperone for the misfolded HGSNAT enzyme. Glucosamine treatment partially restored lysosomal enzymatic activity, which was linked to reduced neuropathology and enhanced short-term memory in the subjects [157]. Additionally, glucosamine analogues, such as peracetylated 4-deoxy-N-acetylglucosamine, are being investigated as candidates for SRT. These molecules work by reducing the synthesis of HS, thereby preventing the excessive accumulation of GAGs. Their efficacy has been demonstrated in various experimental systems, including Drosophila and mouse models, with evidence showing they can effectively be transported across the BBB [158].

In addition to the primary compounds mentioned, other natural modulators such as curcumin, capsaicin, and calcitriol have been evaluated in preclinical tests. In MPS III fibroblasts, these substances demonstrated the ability to lower GAG levels by stimulating lysosomal degradation. However, their therapeutic efficacy was generally found to be lower than that of resveratrol [140].

## 6. New Targets and Therapies

### 6.1. Pharmacological Chaperones

Another therapeutic strategy investigated during the analyzed period is pharmacological chaperone therapy (PCs) [159]. PCs are molecules that connect with high specificity to a target protein and stabilize their native conformation, support the correct folding of the mutant protein, and restore biological activity. PCs for multiple MPS are described. For MPS IIIB, a library of 17 six-membered iminosugar−C−glycosides that imitate the N−acetyl-D-glucosamine semicircle from HS was evaluated. Among them, β-homoiminosugar was found to be the most effective PC, causing a 2.4-fold increase in the activity of the NAGLU mutant at the optimal concentration [159]. Recent studies have shown that a set of N-substituted iminosugars increases NAGLU activity and reduces HS levels in MPS IIIB cellular models. It is important that these molecules can inhibit the synthesis of HS but also act as inactive PC [160].

It was shown by Losada et al. that the docking parameters allow modeling of NAGLU interactions with natural and artificial substrates and can serve as a starting point for identifying potential PC interactions with the active cavity (orthosteric site). In this way, atovaquone and piperaquine were selected as two potential PCs that showed the ability to significantly increase NAGLU activity in MPS IIIB fibroblasts. Although none of the investigated concentrations normalized NAGLU activity, the observed increases in enzyme activity may nevertheless be sufficient to reduce GAG accumulation and potentially offer therapeutic benefits for MPS IIIB patients. Furthermore, the activity increase achieved with these investigated PCs exceeded the values recently reported for MPS IIIB. Pharmacological chaperones appear to be a promising therapeutic strategy due to several advantages. First, they are typically administered orally and may exhibit good absorption from the gastrointestinal tract. Importantly, some compounds have also shown the potential to cross the blood–brain barrier, which is particularly relevant for MPS IIIB, where neurological manifestations are among the most severe aspects of the disease. In addition, they are characterized by lower immunogenicity compared to currently available therapies. Furthermore, even partial restoration of enzyme activity may be sufficient to reduce GAG accumulation and provide a therapeutic benefit. However, the efficacy of pharmacological chaperones is mutation-dependent, meaning that not all variants respond equally well to treatment. In addition, the concentrations required for some compounds to achieve a therapeutic effect may be associated with potential toxicity, highlighting the need for further studies to evaluate their long-term safety and efficacy [159].

However, research is needed to validate their use and safety, particularly in long-term animal studies.

### 6.2. Immunomodulation

Immunomodulatory drugs could represent a relevant medical approach, as the accumulation of undigested substances triggers an innate immune response and promotes inflammatory processes that contribute to disease progression. Unfortunately, immunomodulators are not approved for this indication. For that reason, some researchers are looking for the benefits of therapies to make them widely available for innovative individual treatment (ITT) studies using immunomodulatory care [161].

Two main targets have been identified for intervention in the vicious inflammatory cascade: the upregulation of TLR4 and cytokines/chemokines, and the activation of the NLRP3 inflammasome. Targeting them may be particularly relevant, as accumulated GAGs have been shown to activate these pathways, promoting the release of pro-inflammatory cytokines and contributing to chronic neuroinflammation and disease progression [1]. This led to the selection of nine candidate molecules: adalimumab, anakinra, alemtuzumab, pentosan polysulfate (PPS), ataluren, genistein, cladribine, and odiparcil. Many of these substances have undetermined mechanisms of action. Some of them are able to cross the blood–brain barrier, which may be particularly beneficial in MPS types with significant neurological involvement, whereas others may be more suitable when CNS targeting is not required. In addition, these therapies can be administered through different routes, including oral and subcutaneous administration. Furthermore, certain compounds may induce long-lasting anti-inflammatory effects even after a relatively short treatment course. However, their use may also be associated with adverse effects, including nephrotoxicity, hematological complications, infections, and injection-site reactions [161]. Positive effects of treatment with, among others, Odiparcil have been reported in the ocular phenotypes of MPS IV mouse models [162].

Unfortunately, immunomodulation therapy remains a poorly researched topic. While there are arguments for the positive impact of already discovered modulators, it is first necessary to understand their precise mechanism of action and predict potential adverse effects.

### 6.3. Photobiomodulation

In photobiomodulation therapy (PBM), biological tissue is irradiated with light in the red to near-infrared range (600–1000 nm), thereby improving cellular functions. A chromophore target for PBM is thought to be cytochrome c oxidase complex IV, located in the outer mitochondrial membrane [163]. Following the absorption of photons in the red to near-infrared range, cytochrome c oxidase enables an increased electron flow rate within the mitochondrial electron transport chain across the inner membrane. This process influences the electron availability, creating a proton gradient that improves ATP production, enhancing mitochondrial function. PBM triggers the production of cyclic adenosine monophosphate, which activates transcription factors in neurons that produce cell-protective effects, for example, decreasing microglial activation in the whole brain, reducing IBA1 expression, and restoring their morphology to a more ‘ramified’ state [164].

PBM under infrared light (904 nm) applied to the MPSIII mice model reduced the levels of microglial activation markers and restored their morphology to near normal, reversed the proinflammatory signal of leukocytes, and significantly reduced the number of circulating cytotoxic natural killer cells in the blood, confirming both central and peripheral immunomodulatory effects. Unfortunately, regardless of the light intensity, the accumulation of HS in mouse brain cells was not reduced [165].

Although PBM did not reduce HS levels in cells, which theoretically precludes its use in treating Sanfilippo disease, it may serve as a non-invasive form of therapy to complement conventional treatments such as ERT or stem cell transplantation.

### 6.4. The Use of Lithium Chloride and Valproic Acid

Currently available therapies, such as ERT, reduce somatic symptoms; however, they are not effective in reducing neurological symptoms due to their inability to cross the BBB. The use of lithium chloride (LiCl) and valproic acid (VPA) in treatment appears to be a way to overcome this difficulty.

LiCl and VPA are compounds widely used to treat bipolar disorders. Two frequently described mechanisms of action of LiCl and VPA are the reduction in inositol levels and the inhibition of glycogen synthase kinase 3 (GSK3). The reduction in inositol levels likely explains both the mood-stabilizing effect and other effects, including the stimulation of autophagy. It is also thought that GSK3 inhibition stimulates autophagy.

The ability to stimulate autophagy in cells is the most important factor when considering the use of LiCl and VPA in the treatment of MPS. Stimulation of autophagy in cells of patients with neuropathic forms of MPS (types I, II, III, and VII) with LiCl and VPA led to a significant reduction in the accumulation of GAGs in lysosomes [166]. In light of the evidence presented, therapy with LiCl and VPA may constitute a new, promising form of treatment for neuropathic types of MPS in the future.

## 7. Concluding Remarks

MPS diseases belong to rare conditions that cause significant and severe problems not only to patients but also to their families/caregivers, as was discussed recently [167]. Therefore, the development of effective therapeutic approaches is especially important. On the other hand, the nature of MPS and a complicated pathomechanism make such a task especially difficult. Nevertheless, reports of recent years indicated that there are different possibilities to improve already existing kinds of therapies and develop new ones, to achieve higher efficacy in the treatment of different MPS types. The most intensively developing areas include improvement of ERT (by modifications allowing effective delivery of the therapeutic enzyme to the brain), HSCT (by introducing modulatory elements increasing efficacy), and gene therapy (by developing new, effective gene delivery systems), as well as searching for effective natural compounds and novel targets. One might also consider that combining different therapeutic approaches might give the best results, as any single therapy appears to fail in correcting all MPS symptoms. Combination therapies are receiving increasing attention in MPS research, as they may simultaneously target both the primary disease mechanism and secondary pathological processes. Such approaches may enhance the efficacy of existing treatments, compensate for limitations of individual therapeutic strategies, and provide additional benefits by alleviating disease manifestations that are not sufficiently corrected by a single intervention. This trend can be observed across multiple therapeutic platforms, including, for example, the combination of gene therapy with molecules targeting secondary pathology, as well as RNA-based approaches combined with compounds that enhance their therapeutic efficacy. Another important aspect is the identification of biomarkers that may help evaluate whether a therapeutic intervention is effective. These include glycosaminoglycan levels in urine, plasma, and cerebrospinal fluid. Furthermore, enzyme activity measurements may serve as valuable biomarkers, particularly in therapies designed to restore or increase the activity of the deficient enzyme [161]. An additional challenge in MPS management is the cost and availability of therapeutic interventions. ERT is associated with particularly high costs due to the need for lifelong repeated administration, whereas HSCT, despite being expensive, is performed as a one-time procedure. Although gene therapies for MPS are not yet widely available, the high costs of currently approved gene therapies suggest that future gene-based treatments may also pose substantial economic challenges [168]. An additional challenge is the availability of therapies, which are not always uniform across all regions and patient groups. Access to treatment may vary depending on the MPS type, patient age, and local healthcare infrastructure. Consequently, some patients may experience greater difficulties in obtaining appropriate therapy than others [169,170]. 

Finally, it appears clear that early starting any kind of therapy for MPS provides a significantly higher chance for its efficacy than under conditions where a therapy is initiated at a late stage of the disease. Some changes might be irreversible at that time; thus, special efforts for early diagnosis and early therapeutic interventions are especially important. In fact, the molecular mechanisms of MPS are complex and result in severe clinical manifestations, thus requiring different therapeutic approaches. Importantly, understanding details of the pathomechanism of the disease and the molecular bases of clinical symptoms’ appearance allows proposing and testing various methods of MPS treatment. Despite only partial improvement being possible to date, deeper knowledge on specific molecular transactions occurring in MPS should facilitate developing more efficacious therapies and/or their combination to achieve satisfactory clinical effects.

## Figures and Tables

**Table 1:** Summary of clinical, genetic, and therapeutic characteristics of MPS.

MPS Type	Eponym and Subtype	OMIM Number	Gene	Enzyme	GAG(s)	Major Symptoms	Currently Available Therapies	Reference(s)
MPS I	Hurler syndrome (MPS IH)	607014	*IDUA*	α-L-iduronidase	HS, DS	Multisystem abnormalities with neurocognitive symptoms in MPS IH (sometimes also in MPS IH/S)	ERT (laronidase [Aldurazyme^®^]), HSCT	[3,8,9]
	Hurler/Scheie syndrome (MPS IH/S)	607015						
	Scheie syndrome (MPS IS)	607016						
MPS II	Hunter syndrome	309900	*IDS*	iduronate-2-sulfatase	HS, DS	Neurocognitive (in most cases) and multisystem abnormalities	ERT (idursulfase [Elaprase^®^], pabinafusp alfa [IZCARGO], tividenofusp alfa [AVLAYAH])	[8,10,11]
MPS III	Sanfilippo syndrome type A	252900	SGSH	heparan-N-sulfatase	HS	Especially severe neurocognitive and milder multisystem abnormalities	Symptomatic treatment	[8,12,13]
	Sanfilippo syndrome type B	252920	*NAGLU*	α-N-acetyglucosaminidase				
	Sanfilippo syndrome type C	252930	*HGSNAT*	α-glucosaminidase acetyltransferase				
	Sanfilippo syndrome type D	252940	*GNS*	N-acetylglucosamin-6-sulfatase				
	Sanfilippo syndrome type E *	Not available	*ARSG*	Arylsulfatase G		Neurodegenerative changes only in the cerebellum	Not available	
MPS IV	Morquio syndrome A	253000	*GALNS*	N-acetylgalactosamine-6-sulfate sulfatase	KS, C6S	Primarily skeletal, ocular abnormalities	ERT (elosulfase alfa [Vimizim^®^])	[8,11,14]
	Morquio syndrome B	253010	*GLB1*	β-galactosidase	KS		Symptomatic treatment	
MPS VI	Maroteaux-Lamy syndrome	253200	*ARSB*	N-acetylgalactosamine-4-sulfatse	DS, C4S	Skeletal, ocular, hearing, and dental abnormalities, with additional systemic involvement	ERT (galsulfase [Naglazyme^®^])	[8,11,15]
MPS VII	Sly syndrome	253220	*GUSB*	β-glucuronidase	DS, HS, C4S, C6S	Non-immune hydrops fetalis and a multisystem abnormality	ERT (vestronidase alfa-vjbk [Mepsevii^®^])	[8,11,16]
MPS IX	Natowicz syndrome	601492	*HYAL1*	Hyaluronidase 1	HA	Periarticular soft tissue thickening	Symptomatic treatment	[17]
MPS X	-	619698	*ARSK*	Arylsulfatase K	DS	Skeletal, ocular, hearing and heart abnormalities	Symptomatic treatment	[18]
MPS-PS	-	617303	*VPS33A*	VPS33A	HS, DS	Symptoms similar to MPS, but also include congenital heart defects and hematopoietic disorders	Symptomatic treatment	[12,19]
MPS-PS-like	-	Not available	*VPS16*	VPS16	DS	Skeletal, neurocognitive and multisystem abnormalities	Symptomatic treatment	[20,21]

* MPS IIIE has been described only in animal models. Similar to other MPS III types, neurocognitive symptoms are observed, with neurodegenerative changes confined to the cerebellum. However, in humans, pathogenic variants in the ARSG gene cause Usher syndrome type IV, characterized by late-onset hearing loss and retinitis pigmentosa, without any symptoms typical of MPS. Abbreviations: Keratan sulfate (KS); Heparan sulfate (HS); Dermatan sulfate (DS); hyaluronan synthases (HA); Chondroitin 6-Sulfate (C6S); Chondroitin 4-Sulfate (C4S); α-L-iduronidase (IDUA); iduronate-2-sulfatase (IDS); heparan-N-sulfatase (HNS); α-N-acetyglucosaminidase (NAGLU); α-glucosaminidase acetyltransferase (HGSNAT); N-acetylglucosamin-6-sulfatase (GNS); arylsulfatase G (ARSG); N-acetylgalactosamine-6-sulfate sulfatase (GALNS); N-acetylgalactosamine-4-sulfatse (GalNAc4S); β-glucuronidase (βGLU); Hyaluronidase 1 (HYAL1); Hematopoietic Stem Cell Transplantation (HSCT).

**Table 2:** Clinical experience and emerging cellular and gene-based therapeutic strategies in MPS.

MPS Subtype/Model	Therapeutic Approach	Study Type	Key Findings/Relevance for Therapy	Reference(s)
MPS I	HSCT	Clinical—long-term follow-up	HSCT provides durable metabolic correction and improved survival. Early transplantation is associated with better neurological outcomes and remains the standard treatment for severe MPS I.	[41,43,44,45,47,48,53]
MPS II	Early therapeutic intervention and HSCT-related observations	Clinical observations	Early treatment may improve systemic outcomes; neurological involvement remains a major therapeutic challenge.	[49]
Neuronopathic MPS II models	Advanced experimental disease models	Preclinical research	Development of improved models allows evaluation of CNS-targeted gene and cell therapies.	[42,54]
MPS IVA	HSCT	Clinical studies	Early clinical reports suggest potential metabolic benefits but clinical evidence remains limited and impact on skeletal disease requires further investigation.	[50,55]
MPS VI	HSCT in pediatric patients	Clinical studies	Transplantation in early childhood may improve biochemical parameters and slow systemic disease progression.	[51,52]
Multiple MPS models	Gene-modified HSPCs	Preclinical research	Lentiviral-modified HSPCs enable supraphysiological enzyme production and enhanced cross-correction in affected tissues.	[56,57]
Multiple MPS Models	Genome editing (CRISPR-based strategies)	Preclinical research	Targeted gene correction or integration in HSPCs offers a potential one-time curative therapeutic strategy.	[58]
Multiple MPS Models	Optimized therapeutic transgenes	Preclinical research	Engineering of improved transgene constructs enhances enzyme secretion, stability and delivery to difficult-to-reach tissues, including the CNS.	[59]
Multiple MPS Models	Alternative cellular platforms (engineered lymphocytes)	Preclinical research	Modified immune cells may serve as long-term enzyme-producing platforms with improved tissue distribution.	[60]

**Table 3:** Comparison of AAV vectors’ characteristics in MPS gene therapy.

AAV Type	Advantages	Limitations	References
AAV8	stable and high systemic expression, therapeutic effect in children (no significant episomal dilution)	limited CNS and bone penetration, correction often requires higher doses, possible immune response, therapy efficacy dependent on sex efficient correction in hard-to-target tissues required vector optimization and often local delivery, liver-directed therapy alone may be insufficient for effective CNS correction	[84,90,92,95,107,108,109]
AAV9	high systemic expression high tropism: CNS, skeletal and heart muscle and other tissues long-term activity in the brain for up to 7 years possibility of dose reduction when combined with combination therapy	correction often requires higher doses higher efficacy required more invasive CNS delivery (ICV) possible immune response reduced transduction of deep brain structures after CSF delivery efficacy depended on the route of administration long-term joint correction remained limited	[88,89,94,96,98,100,103,104,105,110,111,112,113]
AAVrh.10	high CNS transduction efficiency	local toxicity risk	[85]
rAAV5	long-term therapeutic effect in the CNS behavioral improvement therapy safety	long-term efficacy depended on immunosuppression	[86,87]
scAAV	faster and more efficient transduction, partial CNS correction	reduced transgene packaging capacity results depends on age	[114,115]
AAV8 and AAV2 Capsid-modified	enhanced brain distribution and transduction enabled cross-correction of non-secreted protein	limited translational validation	[91,102,106]

**Table 4:** Comparison of gene therapy approaches used in MPS treatment.

Delivery System or Technology and References	Advantages	Limitations
Lentiviral vectors [76,77,78,79,80,81,82,83]	Long-term transgene expression, no vector dilution effect, transduction of non-dividing cells, controlled cellular modification, reduced off-target effects, reduced immune activation	Conditioning chemotherapy required, treatment-associated adverse effects, complex logistics, specialized laboratory infrastructure, efficacy dependent on early intervention
AAV vectors [84,85,86,87,88,89,90,91,92,93,94,95,96,97,98,99,100,101,102,103,104,105,106,107,108,109,110,111,112,113,114,115]	Episomal persistence; rare genomic integration, lack of replicative capacity, broad serotype diversity, tissue-specific targeting, capsid and transgene modification	Immune responses, local tissue toxicity due to overexpression, delivery-dependent efficacy, dose-dependent efficacy, promoter-dependent efficacy, and route-dependent efficacy
Genome editing technologies [106,116,117,118,119,120,121,122,123]	Gene correction: increased enzyme activity, reduced GAG storage, phenotype correction, reduced lysosomal storage, applicability to monogenic diseases	BBB delivery challenges, unequal tissue distribution, immune responses, off-target effects, safety concerns, further efficacy evaluation required

## Data Availability

No new data were created or analyzed in this study. Data sharing is not applicable to this article.

## Abbreviations

The following abbreviations are used in this manuscript:

AAV	Adeno-associated virus
ARSG	Arylsulfatase
ASO	Antisense oligonucleotides
BBB	Blood–brain barrier
BSID	Bayley Scales of Infant and Toddler Development
C4S	Chondroitin 4-Sulfate
C6S	Chondroitin 6-Sulfate
CRISPR-Cas	Clustered Regularly Interspaced Short Palindromic Repeats- associated protein
CSF	Cerebrospinal fluid
DBS	Double-strand breaks
DS	Dermatan sulfate
EGFR	Epidermal growth factor receptor
ERT	Enzyme replacement therapy
GAGs	Glycosaminoglycans
GalNAc4S	N-acetylgalactosamine-4-sulfatse
GALNS	N-acetylgalactosamine-6-sulfate sulfatase
GNS	N-acetylglucosamin-6-sulfatase
GSK3	Glycogen synthase kinase 3
GVD	Graft-versus-host disease
HA	Hyaluronan synthases
HCAd	High-capacity adenovirus
HGSNAT	α-glucosaminidase acetyltransferase
HNS	heparan-N-sulfatase
HS	Heparan sulfate
HSCT	Hematopoietic Stem Cell Transplantation
hTfR	Human transferrin receptor
HY-AL1	Hyaluronidase 1
ICTV	International Committee on Taxonomy of Viruses
IDS	iduronate-2-sulfatase
IDUA	α-L-iduronidase
ITT	Innovative individual treatment
KS	Keratan sulfate
LDSs	Lysosomal storage diseases
LiCl	Lithium chloride
LV	Lentiviral vector
M6PR	Mannose-6-phosphate receptor
MPS	Mucopolysaccharidoses
NAGLU	α-N-acetyglucosaminidase
NfL	Neurofilament light
NHEJ	Non-homologous end joining
PAM	Potospacer adjacent motif
PBM	Photobiomodulation therapy
PCs	Pharmacological chaperones
PPS	Pentosan polysulfate
PTC	Premature termination codon
SBS	Single-strand breaks
TALENs	Transcription activator-like effector nucleases
TFEB	Transcription factor EB
VPA	Valproic acid
ZFNs	Zinc finger nucleases
βGLU	β-glucuronidase
